# *Xylella fastidiosa* subsp. *pauca* Strains Fb7 and 9a5c from Citrus Display Differential Behavior, Secretome, and Plant Virulence

**DOI:** 10.3390/ijms21186769

**Published:** 2020-09-15

**Authors:** Jessica Brito de Souza, Hebréia Oliveira Almeida-Souza, Paulo Adriano Zaini, Mônica Neli Alves, Aline Gomes de Souza, Paulo Marques Pierry, Aline Maria da Silva, Luiz Ricardo Goulart, Abhaya M. Dandekar, Rafael Nascimento

**Affiliations:** 1 Institute of Biotechnology, Federal University of Uberlandia, Av. Amazonas, Bloco 2E, Campus Umuarama, Uberlandia MG 38400-902, Brazil; souza.jessica@ufu.br (J.B.d.S.); hebreia@yahoo.com.br (H.O.A.-S.); alingosouza@yahoo.com.br (A.G.d.S.); lrgoulart@ufu.br (L.R.G.); rafaelnascimento@ufu.br (R.N.); 2 Department of Plant Sciences, College of Agriculture and Environmental Sciences, University of California, Davis, 1 Shields Ave, Davis, CA 95616, USA; pazaini@ucdavis.edu; 3 Department of Technology, School of Agricultural and Veterinary Studies, São Paulo State University (FCAV/UNESP), Via de Acesso Prof. Paulo Donato Castellane, Jaboticabal SP 14884-900, Brazil; monicanelialves@gmail.com; 4 Citriculture Defense Fund (Fundecitrus), Av. Dr. Adhemar Pereira de Barros 201, Araraquara SP 14807-040, Brazil; 5 Department of Biochemistry, Institute of Chemistry, University of São Paulo, Av. Prof. Lineu Prestes 748, São Paulo SP 05508-000, Brazil; pmpierry@gmail.com (P.M.P.); almsilva@iq.usp.br (A.M.d.S.)

**Keywords:** lipase, hemolysin-like protein, peptidase, phytopathogen, pathogenicity, secretome, plant-pathogen interaction, LC-MS/MS

## Abstract

*Xylella fastidiosa* colonizes the xylem of various cultivated and native plants worldwide. Citrus production in Brazil has been seriously affected, and major commercial varieties remain susceptible to Citrus Variegated Chlorosis (CVC). Collective cellular behaviors such as biofilm formation influence virulence and insect transmission of *X. fastidiosa*. The reference strain 9a5c produces a robust biofilm compared to Fb7 that remains mostly planktonic, and both were isolated from symptomatic citrus trees. This work deepens our understanding of these distinct behaviors at the molecular level, by comparing the cellular and secreted proteomes of these two CVC strains. Out of 1017 identified proteins, 128 showed differential abundance between the two strains. Different protein families were represented such as proteases, hemolysin-like proteins, and lipase/esterases, among others. Here we show that the lipase/esterase LesA is among the most abundant secreted proteins of CVC strains as well, and demonstrate its functionality by complementary activity assays. More severe symptoms were observed in *Nicotiana tabacum* inoculated with strain Fb7 compared to 9a5c. Our results support that systemic symptom development can be accelerated by strains that invest less in biofilm formation and more in plant colonization. This has potential application in modulating the bacterial-plant interaction and reducing disease severity.

## 1. Introduction

*Xylella fastidiosa* is one of the most important phytopathogenic bacteria, causing a variety of economically significant diseases worldwide [1,2]. Among them are citrus variegated chlorosis (CVC) [3], Pierce’s disease of grapevines (PD) [4], leaf scorch diseases in almond (ALS) [5], oleander (OLS) [6], and coffee (CLS) [7] and, the most recent, olive quick decline syndrome (OQDS) described in 2013 in Southern Italy [8,9]. Since then this Gram-negative bacterium has also been detected infecting different hosts in the Mediterranean region [10,11], and strains from subspecies *pauca* have also been recently isolated from olive trees with OQDS symptoms in Brazil [12], confirming its status of emerging pathogen in different regions and crops. Bioeconomic modeling of these emerging diseases calculates devastating economic losses [13]. In Italy, for example, losses ranging from 1.9 to 5.2 billion Euros are projected if production was interrupted due to the eradication of olive trees affected by *X. fastidiosa* subsp. *pauca* [13]. In the case of CVC, *pauca* strains are widespread in all major citrus-producing regions in South America and disease incidence reached over 40% of producing trees in peak years in the early 2000s and since has dramatically been reduced due to a combination of labor and cost-intensive pest control management practices for citrus greening disease [14]. As more *pauca* strains become studied, their common and peculiar genetic characteristics are being described in increasing detail [14,15,16,17]. This information enables a better understanding of intra and inter-subspecies evolution, as well as their relationship with host range and geographic distribution [14]. In light of the rapid dissemination and impact of this phytopathogen, there is great interest in understating the molecular aspects of plant-microbe interactions in these pathosystems. *X. fastidiosa* is known to secrete and respond to diffusible signal factors and perform quorum sensing shifting from a more individual explorer stage to an aggregated community within a biofilm, affecting insect transmission and virulence to plant hosts [18,19,20]. This shift in autoaggregation state is observed in many bacteria that can adapt to changes within and across hosts by employing signaling systems with diffusible signaling factors [21,22].

Gram-negative bacteria commonly employ secretion systems to create a more favorable host microenvironment [23,24,25]. To understand how these pathogens establish an infection, the effectors and functioning of secretion systems can reveal many critical aspects of the pathogen-host interaction [26]. Many extracellular proteins of pathogenic bacteria are determining factors for bacterial virulence and are secreted by different routes [27]. Other systems such as the type III secretion system (T3SS) are capable of injecting effectors directly into host cells and are present in bacterial pathogens of plants and animals [28,29]. Due to the importance of the T3SS, several studies have found molecules capable of inhibiting it, thus reducing the virulence of the pathogen and protecting agricultural production [30,31,32]. However, *X. fastidiosa* differs from this common theme by not presenting this type of secretion system, and consequently not being applicable to the several existing control strategies for bacteria with T3SS [33]. The main secretion systems used by it are types I, II, IV, and V [34,35]. The role of the type I secretion system in *X. fastidiosa*, verified from a mutation in the TolC channel, is indicative of an interference in the pathogenicity and survival of this phytopathogen in the host [36]. The type II secretion system (T2SS) can be considered the most relevant in bacterial pathogenesis [37], and in *X. fastidiosa* most of the proteases and cell wall degrading enzymes (CWDEs) are secreted by this system [38]. In particular, the protein lipase/esterase LesA is a key factor in PD pathobiology [39]. Type IV (*tra* and *trb*) secretion systems are also detected in *X. fastidiosa*, usually in native plasmids [17]. Among the proteins secreted by the type V system, the autotransporter family is the most widespread [40], and in *X. fastidiosa* Temecula1, the XatA autotransporter has been shown to be relevant for the ability of the bacteria to aggregate and for the biofilm formation process [41]. Autotransporter secretion is also performed by XadA trimeric adhesins [42,43]. The two-partner secretion system (also known as Type Vb) is also used by *X. fastidiosa*, delivering large hemagglutinin-like adhesins on the cell surface [44]. Taken together these multiple secretion systems illustrate well the intense trafficking of proteins and metabolites through the bacterial cell wall and their importance in host-microbe interactions.

Surface fimbrial structures that mediate twitching motility and adhesion also rely on porins and usher proteins that promote protein export across the outer membrane during pili assembly, influencing plant colonization and biofilm formation [41,45,46,47]. Outer membrane vesicles (OMVs) are yet another protein export system, also contributing to *X. fastidiosa* pathogenesis [41,48,49,50]. Being much smaller than cells, averaging ~100 nm, some virulence factors can thus be delivered by OMVs to a greater range within the host [39,47]. Among those found in *X. fastidiosa,* OMVs are lipases/esterases, adhesins, proteases, and toxins/antitoxins [39,41,49,50]. Given the importance of these proteins to host-plant interaction, analytical methods such as mass spectrometry can provide a non-biased proteome assessment and improve our understanding of their major functions and specific players. In this study, we compared strains Fb7 and 9a5c of *X. fastidiosa* subsp. *pauca* isolated from symptomatic citrus trees (in Argentina and Brazil, respectively) that displayed a distinct aspect when cultured in vitro: reference strain 9a5c forms a robust biofilm while strain Fb7 remains mostly planktonic even at high cellular density. We further investigated this behavioral difference on a molecular level, performing proteome analyses of total cellular content and secreted samples followed by functional investigation of lipase and esterase activities. Finally, their virulence to *N. tabacum* was compared, confirming that, while both are pathogenic, strain Fb7 is more aggressive and has a distinct molecular profile. Our results contribute to the understanding of *X. fastidiosa* pathogenesis in increasing detail of the protein network involved with both its planktonic and biofilm-forming lifestyles.

## 2. Results

### 2.1. Differential Behavior of X. fastidiosa Strains Fb7 and 9a5c Cultured In Vitro

**Figure 1:**
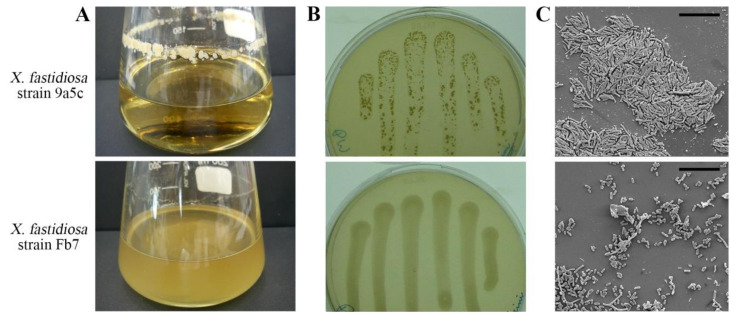
Comparison of aggregation and biofilm formation phenotypes of *Xylella fastidiosa* strains 9a5c and Fb7. (**A**) The reference strain 9a5c forms large aggregates and compact biofilm structures, while strain Fb7 remains mostly planktonic and forms very little biofilm on glass under agitation. (**B**) Cellular suspensions spotted on PW agar plates and drained to the other side of the plates forming the columns also show a distinct biofilm formation between the strains after 7 days of growth, with a wax-like texture for 9a5c and a gum-like texture for Fb7. (**C**) Scanning electron micrographs of glass coverslips observed after 3 days in bacterial cultures show the distinct aggregation behavior between the strains at the cellular level. Scale bar is 10 µm for both micrographs.

The reference strain 9a5c has become a model for *X. fastidiosa* studies on citrus since the sequencing of its genome [34]. It presents autoaggregation and intense biofilm formation when cultured in vitro [51,52]. Strain Fb7 was later used for microarray-based genome comparisons and more recently had its genome completely sequenced as well, and used in proteome and lipidome study of OMVs [16,17,49]. This strain displays a characteristic aspect when cultured in vitro, not forming cellular aggregates as seen for strain 9a5c, both in broth and on solid media (Figure 1A,B). A closer inspection of initial stages of bacterial microcolonies forming on glass surfaces also shows the differential autoaggregation behavior between strains Fb7 and 9a5c (Figure 1C).

### 2.2. Proteomics of X. fastidiosa Strains Fb7 and 9a5c

**Figure 2:**
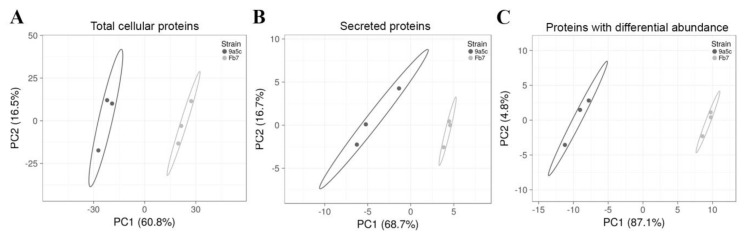
Clustering of samples used in proteomics by principal component analysis (PCA). Effect on principal components and tightness of clusters is seen when (**A**) all 1011 detected proteins in the total cellular extracts are used in the clustering or (**B**) when 18 proteins detected in the secretome are used or (**C**) when the 128 proteins with differential abundance are used, forming tighter clusters. Samples from strain 9a5c consistently clustered on the left side of the plots, while those from strain Fb7 on the right side of the plots. Prediction ellipses are such that with probability 0.95, a new observation from the same group will fall inside the ellipse.

To better understand the distinct in vitro behaviors regarding biofilm formation and motility of *X. fastidiosa* strains Fb7 and 9a5c, protein samples obtained from pelleted broth cultures (total cellular extracts) and supernatant (secreted extracts) from both strains were compared. Liquid chromatography coupled to mass spectrometry (LC-MS/MS) revealed a total of 1011 *X. fastidiosa* proteins in the cellular extracts passing our quality control filters of 0.1% false discovery rate (FDR) for peptides, 1% FDR for proteins, and a minimum of 2 peptides mapped per protein (Supplementary Materials Table S1). Eighteen proteins were detected in the secreted samples (Table S2). Proteins with differential abundance between the strains (*p*-value < 0.05 and FDR < 0.05) summed 119 in the total cellular extracts and 16 in the secreted extracts (Table S3). Protein samples were grouped by principal component analysis (Figure 2), forming distinct clusters based on sample type (bacterial strains). A correlation matrix of replicate samples by type shows a minimum of 0.936 for Fb7 and 0.962 for 9a5c considering total cellular extracts and 0.908 and 0.834 for secreted extracts of Fb7 and 9a5c, respectively (Table S4).

**Figure 3:**
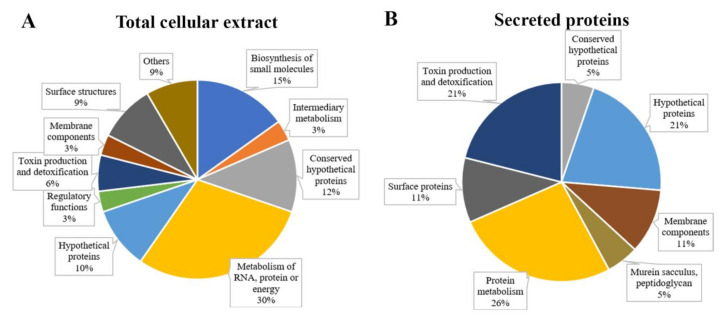
Functional distribution of identified *X. fastidiosa* proteins. Total cellular extract proteins (**A**) and secreted proteins (**B**) were grouped according to their biological processes and are expressed in percentage of the total proteins identified.

**Figure 4:**
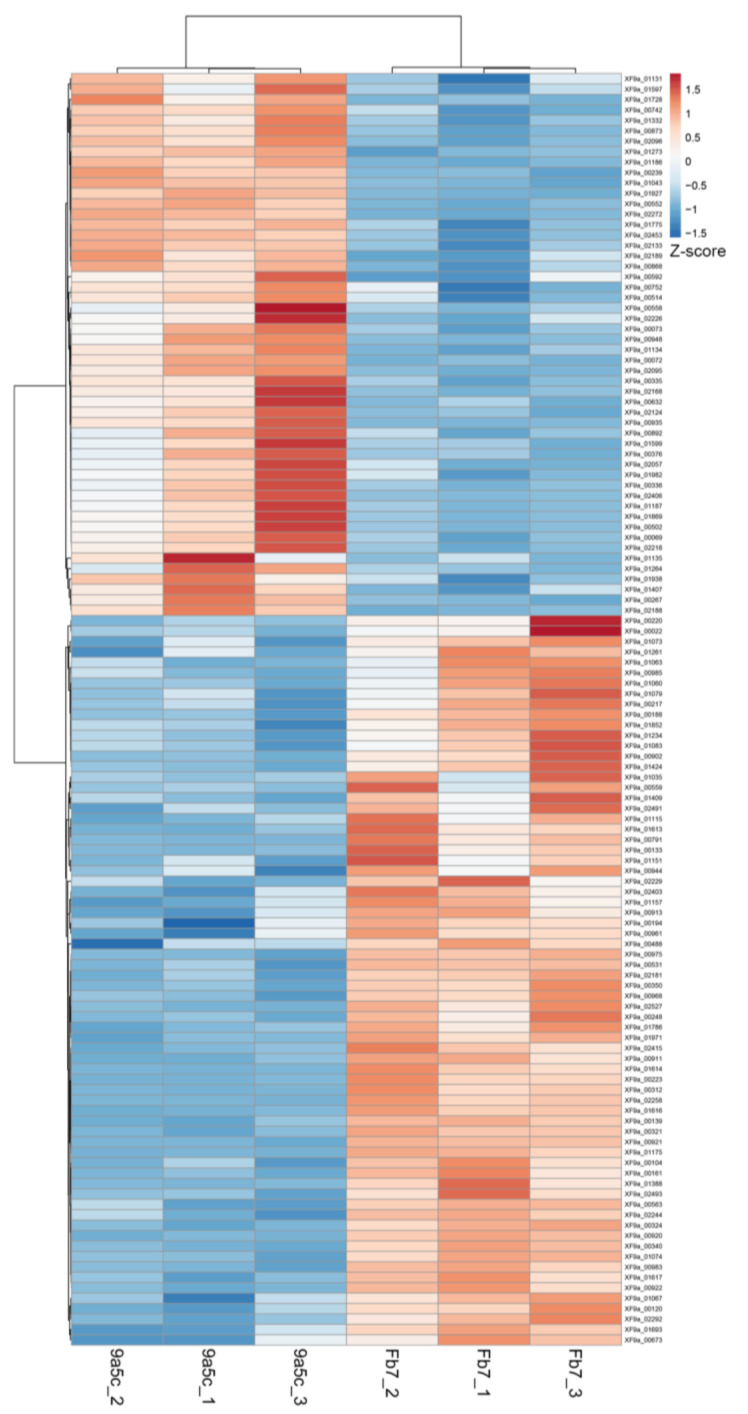
Heatmap showing clustering of samples used for proteomics based on proteins with differential abundance. Comparison of total cellular extracts of *X. fastidiosa* strains Fb7 and 9a5c revealed these 119 proteins. Z-scores of protein abundances show the number of standard deviations from average values. Both rows and columns are clustered using correlation distance and average linkage. Full list is presented in Table S4, which was constructed from Table S1.

The top 20 proteins with significant differential abundance (69 in strain Fb7 and 50 in 9a5c; *p* < 0.05 Benjamin–Hochberg permutation test) are listed in Table 1 and Table 2 (complete list in Table S3). Secreted proteins with differential abundance are listed in Table 3. Proteins with unknown function in the original annotation were manually curated using the UniProt and JGI-IMG Knowledgebases to update the annotation when available. Despite this effort, 22% of the identified proteins remain without a functional annotation. Functional analysis of identified proteins with annotated domains shows a strong contribution of primary metabolism but also biosynthesis of small molecules, surface structures, toxin production, and detoxification (Figure 3A). Among the secreted proteins, most are hydrolases acting to modify the bacterial microenvironment (Figure 3B). Network analysis demonstrated the functional associations between the proteins obtained in the total and secreted samples (Figures S1 and S2). Functional enrichment analysis of gene ontology terms (cellular component, molecular function, and biological process) and KEGG pathways is presented in Table S5. This analysis highlights biosynthesis of secondary metabolites, antibiotics, and arginine metabolism as features that are strongly detected in the proteome. One hundred and nineteen proteins presented differential abundance between strains Fb7 and 9a5c, also enabling clustering of samples based on the bacterial strain (Figure 4).

In the total cellular protein extract, some of the proteins with the highest abundance in both strains include the A subunit of DNA topoisomerase IV encoded by (XF9a_01235/XF1353, these are JGI-IMG and LBI identifiers, respectively), the cell division topological specificity factor MinE (XF9a_01209/XF1320), and, as expected, ribosomal and oxireductases. The outer membrane lipoprotein-sorting protein (XF9a_01326/XF1452) and the ABC-type transport system component (XF9a_00376/XF0418) also stand out, suggesting intense cell wall remodeling and secretion activity by both strains. The hypothetical proteins XF9a_00636, XF9a_02095, XF9a_01194/XF1305 also figure among the most abundant in both strains and should be investigated in more detail to reveal their precise function. Sixty-nine proteins with higher abundance in strain Fb7 of *X. fastidiosa* include the autolytic lysozyme (XF9a_01489/XF2392), further reinforcing intense cell wall remodeling. Additional outer membrane proteins such as OmpW (XF9a_00791/XF0872), the trimeric autotransporter adhesin XadA1 (XF9a_01388/XF1516), the TonB-dependent receptor (XF9a_00312/XF0339), and the multicopper oxidase (XF9a_02527/XF2677) are also more abundant in Fb7 preparations. These samples also included a few proteases/peptidases, such as XF9a_00139/XF0156, XF9a_01175/XF1282, XF9a_02415/XF2551, and XF9a_00120/XF0138. The proteome results also show higher abundance of the gluconolactonase XF9a_01614/XF1742 in strain Fb7 extracts acting on a wide range of hexose-1,5-lactones and secondary metabolites according to the KEGG annotation.

Arginine metabolism also seems more active in Fb7 than in 9a5c as ornithine carbamoyltransferase (XF9a_00920/XF0998), argininosuccinate synthase (XF9a_00921/XF0999), acetylornithine deacetylase (XF9a_00922/XF1000), and arginine deaminase (XF9a_01151/XF1250) are also more abundant. Strain Fb7 also displays a higher activity of the oxidative branch of the pentose phosphate pathway with enzymes including glucose-6-phosphate 1-dehydrogenase (XF9a_00985/XF1065) and 6-phosphogluconolactonase (XF9a_00983/XF1063). Daunorubicin C-13 ketoreductase encoded by (XF9a_01613/XF1741) is also higher in Fb7, catalyzing the synthesis of an antibiotic. Malate oxidoreductase (XF9a_00902/XF0977) was also detected in higher levels in Fb7 and might affect generation of hydrogen peroxide. The lipase/esterase LesB encoded by (XF9a_00324/XF0358) was also more abundant in the total cellular extract of strain Fb7, as well as in the secreted samples. Besides LesB, LesA (XF9a_00323/XF0357) was also among the proteins with higher abundance in the secretome of strain Fb7 (Table 3). The cysteine/serine peptidase (XF9a_01310/XF1434) and the trimeric adhesin XadA1 mentioned before was also detected at higher levels in the secretome of this strain.

The total cellular extract of strain 9a5c showed higher abundance for 50 proteins. Among the most prominent are the hemolysin-like repeats-in-toxin (RTX) cytotoxin (XF9a_02272/XF2407), serine protease PspB (XF9a_00948/XF1026), and Zn-dependent peptidase (XF9a_00742/XF0816). Several type IV pilus components including pilins (XF9a_02403/XF2539 and XF9a_02406/XF2542), and assembly proteins PilO (XF9a_00335/XF0371) and PilP (XF9a_00336/XF0372) show higher abundance in this strain, besides the type I pilus adhesin MrkD (XF9a_00069/XF0078) and usher protein FimD (XF9a_00072/XF0081). These structures are important for surface adhesion and microcolony organization, the initial stages of biofilm formation [45]. Another interesting difference between the two investigated strains is the higher level of the TenA family transcriptional regulator (XF9a_01927/XF2041). Considering the secreted proteins, again the RTX toxins stand out, represented by XF2407 also detected in higher levels in the total cellular extract, plus XF9a_00936/XF1011 and XF9a_00602/XF0668. Strain 9a5c showed higher levels of the autotransporter trimeric adhesin XadA3 (XF9a_01869/XF1981). Perhaps the most intriguing result in the secretome of strain 9a5c is the 8-fold higher content of the conserved protein of unknown function XF9a_01552/XF1577 compared to strain Fb7. This protein has recently been detected in OMV-enriched samples of 9a5c, but not in Fb7 ones [49]. Another protein of unknown function with higher levels in the 9a5c secretome is (XF9a_01177/XF1287). Curiously, this hypothetical protein has only been found in *Xylella* spp. (present exclusively in 52 *X. fastidiosa* genomes, searching against the whole JGI-IMG database, as of August 2020) and here we show its detection. Thus, the hypothetical protein encoded by XF9a_01177/XF128 deserves further characterization. The serine protease PspB, XF9a_00245/XF0267, and the cysteine/serine protease XF9a_00488/XF0531 were also more abundant in the secretome of strain 9a5c. XF0267 was not detected previously in the OMV-enriched fractions [49] and might be exclusive to the OMV-independent fraction. Elongation factor Tu encoded by XF9a_02482/XF2628 is also worth mentioning as, although it displayed similar levels between both strains, it is highly abundant in their secretome, possibly triggering responses to pathogen-associated molecular patterns (PAMP) in the plant host.

### 2.3. Quantitative Real-Time PCR (RT-qPCR)

**Figure 5:**
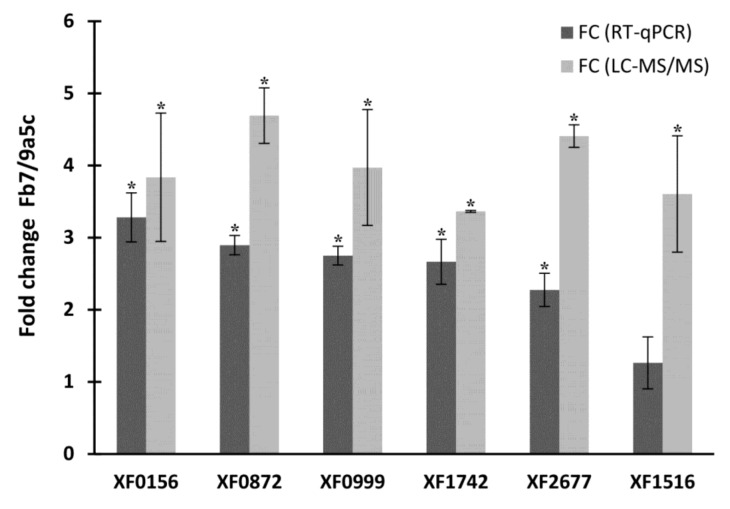
Expression ratios of selected genes comparing *X. fastidiosa* strains Fb7 and 9a5c, obtained by transcript (RT-qPCR) and protein abundance (LC-MS/MS). Results are expressed as mean ±SD and considered significant (*) if *p* < 0.001, Dunnett’s multiple comparison for RT-qPCR data from three biological replicates assayed in three technical replicates each, and *p* < 0.05, Benjamin–Hochberg permutation test for LC-MS/MS data from three biological replicates.

Some of the main proteins identified with higher abundance in strain Fb7 by LC-MS/MS (XF0872; XF1742; XF2677; XF0156; XF0999; XF1516) were also investigated at the transcriptional level by RT-qPCR to verify the correlation between transcript levels and their protein products. Among the six protein-coding genes evaluated, five also presented significant (*p* < 0.001 Dunnet’s multiple comparison) higher levels of expression in strain Fb7 when compared to strain 9a5c (Figure 5), confirming the trends obtained by proteomics also at the transcript level. The only exception was XF1516, for which the difference observed in RT-qPCR results between the strains was not significant.

### 2.4. Esterase and Lipase Activities

**Figure 6:**
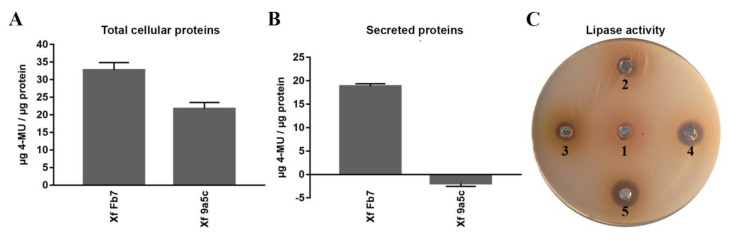
Esterase and lipase activities of *X. fastidiosa* strains Fb7 and 9a5c protein extracts. Esterase activity of (**A**) total cellular and (**B**) secreted proteins. (**C**) Lipase activity of the proteins secreted from *X. fastidiosa* strain 9a5c at 6 and 10 days of culture (2 and 3) and *X. fastidiosa* strain Fb7 at 6 and 10 days of culture (4 and 5). The PW culture medium was used as a negative control (1).

The protein LesA was one of the most expressed in *X. fastidiosa* Fb7 secretome compared to strain 9a5c (Table 3). Having knowledge of its importance for virulence of *X. fastidiosa* [39], enzyme assays were performed to compare the esterase and lipase activities in the protein samples of both strains. In the esterase activity, the quantification was performed by measuring the amount of 4-methylumbeliferone (4-MU) generated from the 4-methylumbeliferyl butyrate (4-MUB) substrate. Evaluation of the total cellular extract and secreted proteins showed a higher capacity of degradation of short-chain esters by strain Fb7 over 9a5c, both in the total cellular extract and secreted samples (Figure 6A,B). To evaluate the lipase activity, a tributyrin substrate was emulsified and incorporated in the agarose gel. The results were similar to the previously described assay, in which a greater zone of clearance forming a halo caused by the degradation of the emulsion due to lipase activity was seen in strain Fb7 when compared to strain 9a5c (Figure 6C). This observed activity validated the greater lipase activity in samples from bacterial strain Fb7, as observed in the proteomic results.

### 2.5. In Vivo Plant Infection Assay

**Figure 7:**
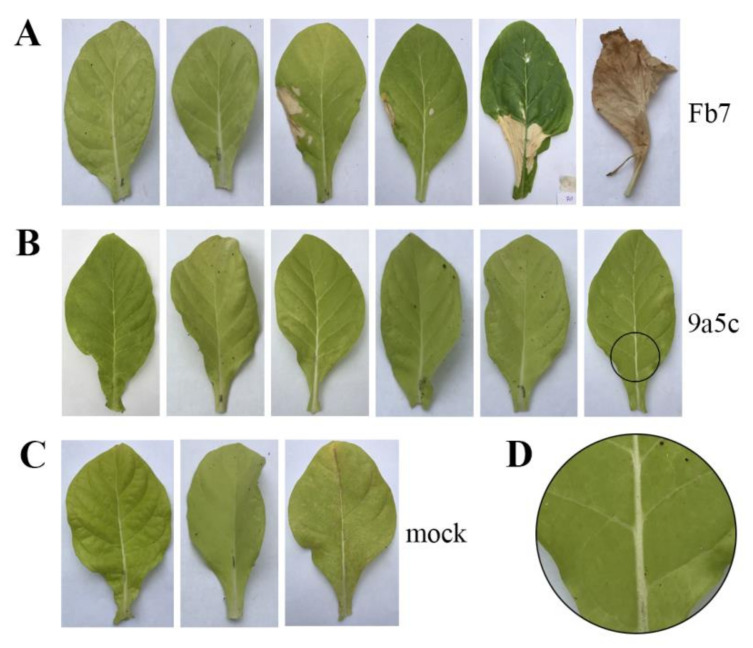
*Nicotiana tabacum* leaves, after 30 days of inoculation with *Xylella fastidiosa* strain Fb7 (**A**), *Xylella fastidiosa* strain 9a5c (**B**), and the negative control (**C**) inoculated with the PW medium (mock). These are representative of the most severe symptoms observed. (**D**) Close-up showing veinal chlorosis.

To visualize the disease response between *X. fastidiosa* strains Fb7 and 9a5c, individual leaves of *N. tabacum* plants were inoculated on petioles and evaluated daily to determine the virulence response based on leaf symptoms. After 30 days of inoculation, leaves showing veinal chlorosis totaled 68% for strain Fb7 and 58% for strain 9a5c, which is not a significant difference in a direct comparison (*t*-test *p* < 0.514). However, unlike strain 9a5c, leaves inoculated with strain Fb7 showed additional symptoms. Intense darkening was observed in 11% of the leaves and 21% developed scorching in part or the complete leaf. Considering these additional symptoms, the difference between the two strains is more evident and the most severe symptoms observed in each group are shown in Figure 7.

## 3. Discussion

*X. fastidiosa* inhabits xylem vessels and mouthparts of sap-feeding insect vectors, two dynamic microenvironments that require the ability to coordinate strong attachment to surfaces with enough motility to cope with population growth under favorable conditions. This bacterium has adapted a tunable collective behavior responsive to diffusible signaling factors that switch individual motile cells into sessile aggregates maturing into biofilms [53,54]. Despite being devoid of a type III secretion system, virulence and pathogenicity mechanisms of *X. fastidiosa* are quite complex and also affected by quorum sensing mechanisms [46]. As new *X. fastidiosa* isolates became available, we noticed differential in vitro biofilm formation among them. Given the demonstrated importance of this collective behavior to disease development, here we compared in further detail two of the most extreme behaviors in our collection, the reference CVC strain 9a5c that forms a prominent biofilm and strain Fb7 that remains mostly planktonic even at high population densities. Both strains are derived from subspecies *pauca* and were isolated from symptomatic citrus trees, which eliminates many potential sources of noise from the comparison. Besides a differential behavior in in vitro culture, these strains also show marked differences in their proteome, both in their total cellular extract and secretomes. This analysis confirmed previously identified and characterized virulence factors such as lipase/esterases [39], as well as other less studied factors such as proteases and RTX proteins. The proteome data corroborated the transcriptional analysis of selected genes, and the lipase and esterase activities of the extracted proteins were further confirmed to validate the proteome results. Finally, the two strains were compared in the model tobacco plant, which allows a quicker evaluation of the symptom response in comparison to their original citrus host that can take years to develop visual symptoms.

By analyzing the proteins from total cellular and secreted extracts, it was possible to verify the presence of proteins relevant for the virulence of *X. fastidiosa* in both strains. Among the most expressed proteins in strain Fb7, an outer membrane receptor similar to OmpW stands out. It is a known virulence and pathogenicity factor in other Gram-negative bacteria and also a receptor for colicin S4 [55,56,57]. Another highly abundant protein in the total cellular extracts is a member of the SMP-30/gluconolactonase/LRE family protein encoded by XF1742. The same genomic vicinity also encodes other members with higher levels in Fb7, including an oxidoreductase, a Daunorubicin C-13 ketoreductase, and an alpha/beta-hydrolase. In *Pseudomonas syringae*, the gluconolactonase ortholog is responsible for modulating quorum sensing [58]. Quorum sensing plays an important role in *X. fastidiosa* pathobiology [20], but the precise contribution of this protein remains to be further analyzed. In *X. fastidiosa* strain Temecula1, a mutant of the *tonB* homolog, *tonB1*, showed decreased biofilm formation and twitching motility, in addition to causing attenuated PD symptoms [59]. We also detected enzymes involved in nitrogen metabolism. In *Xanthomonas oryzae,* argininosuccinate synthase *argG* is important for its growth and pathogenicity with its inactivation causing metabolic defects and compromised growth and virulence [60]. The enzyme L-ascorbate oxidase is responsible for the conversion of ascorbate into monodehydroascorbate [61] where it is involved in several physiological processes as plant growth and development, reduction/oxidation (redox) regulation in the extracellular matrix, and response to abiotic stresses [62,63]. In addition, the oxidation of ascorbate, performed by the enzyme, provides protection against the rice root-knot nematode [64]. The silencing of the gene encoding an alternative oxidoreductase (NADH: ubiquinone oxidoreductase) interfered with the pathogenesis of *Rhizoctonia solani* in tomato and rice [65].

Among the proteins with higher abundance in strain 9a5c, fimbrial proteins, an ortholog of the BtuB cobalamin receptor, and an ortholog of the serine protease PspB were most prominent. The fimbrial proteins are structural components of type I and type IV pili. In *X. fastidiosa,* such structures are associated with surface adhesion, increased aggregation, biofilm formation, and motility [66,67,68]. Their contributions to virulence of this bacteria have been demonstrated since mutations associated with type IV pili showed changes in its phenotype, such as the absence of long pili and lack of twitching motility [45,69,70]. Our data reinforce the role of type IV pilus in biofilm formation and not simply motility. The other proteins highlighted in this work include the serine protease PspB. It has also been identified in other in vitro assays of *X. fastidiosa*, and is absent in another *pauca* strain J1a12 that is not able to induce CVC symptoms in controlled inoculations [51,71]. In addition, secretion of this protease can be important in the dissemination of the bacteria in the host [72]. Many uncharacterized proteins obtained by proteomics need to be analyzed and studied in more detail to elucidate their functions. The interaction network (Figure S1) shows functional associations of some of them with the serine protease PspB, trimeric adhesin XadA1, and outer membrane protein assembly factor BamD. Since these proteins are already characterized and some of them have known importance in the infection process, they may thus present some similarity or interaction with the uncharacterized ones and support formulation of hypotheses about their roles.

Most of the proteins found in the secreted samples (Table 3) were also detected in samples enriched with outer membrane vesicles of *X. fastidiosa* [49]. This secretion strategy is known to also interfere with adherence to surfaces besides delivering virulence factors to sites within the plant that are distant from the producing bacterial cells [39,73]. Peptidases are important proteins in several physiological processes, such as the control of protein functionality throughout development and defense [74,75,76,77]. Strain 9a5c shows higher abundance of proteases, membrane-associated proteins, and toxins. In addition to the importance of the serine protease PspB mentioned above, the relevance of this class is demonstrated by another secreted protease, PrtA, that is known to influence biofilm formation, motility, cell length, and virulence of this phytopathogen in grapevines [78]. Another interesting group of proteins with high abundance include the hemolysin-like RTX proteins. These pathogenicity factors are present in many Gram-negative bacteria and subgroups perform different functions [79,80]. Here, we demonstrate their differential expression in strains with differential biofilm formation. Similar RTX proteins that have been characterized in more detail require relatively high calcium levels, in the millimolar range, to properly fold and to acquire biological activity [79]. It is also known that calcium levels exert a strong influence on *X. fastidiosa* biofilm formation [81]. The relationship of biofilm/planktonic cells behavior and the speed and severity of symptom development has also been observed [19,20,44]. Given the multiple functions associated with RTX proteins, whether these highlighted in the secretome of *X. fastidiosa* are truly bacteriocins or have adapted to other functions, perhaps playing a structural role in biofilm maturation, remains to be demonstrated.

Some proteins highlighted in proteome data were also investigated at the transcriptional level to see how well these two stages of expression are correlated. The validation by RT-qPCR indicated a higher expression of most genes evaluated in strain Fb7 when compared to strain 9a5c, corroborating with the results presented in the proteomics tables. The existing variation in fold-change (FC) values may occur between one technique and another because they use different targets in the analysis (protein and cDNA). In particular, XadA1 XF1516 shows a higher difference in the proteomic than the transcriptomic data. This might reflect a greater difference in accumulation rather than synthesis, as strain Fb7 is known to produce a greater amount of outer membrane vesicles, where this protein is most abundant [49,73].

Given that LesA was detected in the secretome, we compared the esterase and lipase activities in protein extracts of the different strains. Strain Fb7 presented higher activities than strain 9a5c in both assays, as predicted from proteomic results. The measurement of the esterase activity is possible by the presence of the highly fluorescent compound, 4-MU (4-methylumbelilferone) [82]. This is generated by the action of esterases that catalyze the hydrolysis of the fatty acid molecule ester bond, which is contained in the compound 4-MUB (4-methylumbeliferyl butyrate) [83]. Assessment of the lipase activity was carried out by means of the visualization of the halos, resulting from the hydrolysis of tributyrin (C4) by the lipases that release one molecule of glycerol and three molecules of butyric acid [84].

The evaluation of Fb7 and 9a5c strains infectivity in *N. tabacum* presented symptoms of chlorosis on leaves, characteristic of this pathosystem [85]. In addition, some leaves inoculated with strain Fb7 displayed even more evident characteristic symptoms of leaf scorch and tissue darkening [86,87]. This may be due to the higher concentration of some of the proteins mentioned above or in addition to a stronger host response to the bacterial cells or its secretion products. *N. tabacum* is considered an important host for experimental trials since it presents susceptibility to the CVC pathogen and faster development of symptoms when compared to citrus [85]. However, although it is a model plant for evaluating pathogen-host interactions [88], complementary trials in the natural host could strengthen the comparison provided herein. In addition, the effects of these different characteristics revealed here on the interaction with insect vectors and consequently transmission between plants should also be investigated.

In conclusion, proteome studies of *X. fastidiosa* are providing a more complete molecular picture of its different strategies of interaction with the host, revealing major players related to pathogenicity, virulence, and bacterial survival in vivo [39,48,49]. Detection of specific proteins even with unknown functions fosters hypotheses about their roles in the mechanisms of infection and defense, helping in the development of control and therapeutic strategies. Powerful unbiased methods such proteome analyses by LC-MS/MS enable assessment of molecular players that can help us understand the different phenotypes exhibited by these *X. fastidiosa* strains. Complementation with other methods and pathogenicity assays in a model plant supported the notion that formation of stronger cellular aggregates and a more compact biofilm by strain 9a5c results in slower systemic disease progression. In contrast, the phenomenon of hypervirulence triggered by the bacterial population aggregation state has been previously observed [44,78,89]. Indeed practical approaches in controlling bacterial aggregation and motility *in planta* have shown promising results in reducing disease symptoms [19,20], which can be complemented by targeting specific proteins highlighted in the proteome analyses.

## 4. Materials and Methods

### 4.1. X. fastidiosa Strains and Growth Conditions

*X. fastidiosa* CVC strains Fb7 [15,16] and 9a5c [34,90] were routinely cultured for 7 days at 28 °C in periwinkle wilt agar medium supplemented with 0.5% glucose and devoid of albumin (PWG) [49] modified from the original PW [5]. Strain Fb7 was obtained from Dr. Helvecio D. Coletta-Filho, IAC, Brazil. To obtain liquid bacterial cultures, cells were harvested from the agar plates and inoculated in PW broth with optical densities at 600 nm (OD_600_) of 0.05. Cultures were maintained for up to 10 days at 28 °C in a rotary shaker at 200 rotations per minute. Samples to be analyzed by scanning electron microscopy (SEM) were prepared according to Rodrigues et al. [91] and analyzed at CEMI, Unifesp, Brazil.

### 4.2. Preparation of Total and Secreted Protein Extracts from X. fastidiosa

After 7 days of growth, three individual cultures of *X. fastidiosa* strains Fb7 and 9a5c were centrifuged (8000 rpm, 20 min, 4 °C) and the supernatants concentrated (3 kDa filters—AMICON ULTRA-15, Millipore Sigma, Saint Louis, MO, USA), constituting the secreted protein extracts. To obtain the total protein extracts, the pellets were extracted with BugBuster^®^ (Millipore Sigma, Saint Louis, MO, USA) added with protease inhibitors (5 µM phenylmethylsulfonyl fluoride (PMSF), 5 µM benzamidine and 0.5 mM EDTA). The secreted and total cellular protein extracts were precipitated with trichloroacetic acid (TCA)/acetone (1:8:1 sample/acetone/TCA) at −20 °C, 1 h. Then, the pellets were centrifuged (5000 rpm, 30 min, 4 °C) and suspended (2 M Thiourea, 40 mM Tris, 2% 3-[(3-Cholamidopropyl)dimethyl-ammonio]-1-propane sulfonate (CHAPS), 18 mM DTT). Secreted and total cellular protein content was determined using Bradford reagent (Bio-Rad Laboratories, Hercules, CA, USA) according to manufacturer’s instructions.

### 4.3. Proteomic and In Silico Analysis

Three hundred micrograms (300 µg) of three independent secreted and total protein samples for each *X. fastidiosa* strain were precipitated with four times the volume of ProteoExtract Protein Precipitation Kit (Calbiochem, now part of Millipore Sigma, Saint Louis, MO, USA) according to the manufacturer’s instructions. The samples were reconstituted in 100 µL of 6 M urea in 50 mM triethylammonium bicarbonate (TEAB) plus 5 mM dithiothreitol (DTT) and incubated at 37 °C for 30 min. Subsequently, 15 mM Iodoacetoamide (IAA) was added followed by incubation at room temperature for 30 min. The IAA was then neutralized with 30 mM DTT during 10 min. Lys-C/trypsin was added (1:25 enzyme/total protein) and incubated at 37 °C for 4 h. Then, 550 µL of 50 mM TEAB was added to dilute the urea and activate trypsin digestion overnight at 37 °C. The digested peptides were desalted with Aspire RP30 Desalting Tips (Thermo Fisher Scientific, Waltham, MA, USA) and 80 ug of each sample was labeled using TMTsixplex™ Isobaric Label Reagent Set (Thermo Fisher Scientific, Waltham, MA, USA). Twenty micrograms of each labeled sample were combined and fractionated by polarity. One microgram of each fraction was resuspended in 0.1% TFA (trifluoroacetic acid) plus 2% ACN (acetonitrile) and injected sequentially. Peptides were analyzed on a QExactive mass spectrometer (Thermo Fisher Scientific, Waltham, MA, USA) coupled with an Easy-LC source (Thermo Fisher Scientific, Waltham, MA, USA) and a nanospray ionization source. The peptides were loaded onto a Trap (100 µm; C18 100-Å 5U) and desalted online before separation using a reversed-phase (75 µm; C18 200-Å 3U) column. The duration of the peptide separation gradient was 60 min using 0.1% formic acid and 100% acetonitrile (ACN) for solvents A and B, respectively. The data were acquired using a data-dependent MS/MS (tandem mass spectrometry) method, which had a full scan range of 300 to 1600 Da and a resolution of 70,000. The resolution of the MS/MS method was 17,500, and the insulation width was 2 m/z, with normalized collision energy of 27. The nanospray source was operated using a spray voltage of 2.2 kV and a transfer capillary temperature heated to 250 °C. The raw data were analyzed using X!Tandem and viewed using the Scaffold Software v. 3.01 (Proteome Software, Portland, OR, USA). Samples were searched against *Xylella fastidiosa* LBI and JGI-IMG databases appended with the cRAP database, which recognizes common laboratory contaminants. Network analysis was carried out using STRING version 11.0 using *X. fastidiosa* 9a5c as the reference organism [92]. Sample and protein cluster analysis was performed using ClustVis [93].

### 4.4. Real-Time Quantitative PCR (RT-qPCR)

The primers used in this study are listed in Table S6, and their targets were the genes encoding the proteins with the highest Fb7/9a5c fold change values according to the proteomic results. Total RNA (including ribosomal RNA) was extracted (PureLink RNA Mini Kit—Ambion, Waltham, MA, USA) from three independent samples of bacterial cultures of *X. fastidiosa* Fb7 and 9a5c and treated with RQ1 DNase (Promega, Madison, WI, USA). One microgram of treated RNA was used to synthesize first-strand cDNA using the reverse transcriptase M-MLV kit (Thermo Fisher Scientific, Waltham, MA, USA), according to the manufacturer’s instructions in a Mycycler thermocycler (Thermo Fisher Scientific, Waltham, MA, USA). Briefly, a 20 µL reaction mixture was assembled to contain 1× RT buffer, 0.5 mM dNTP, 100 ng of random primer (Invitrogen, Waltham, MA, USA), 0.5 µM *X. fastidiosa* 16S forward primer, 10 units of RNase inhibitor (Invitrogen, Waltham, MA, USA), 4 units of Qiagen Omniscript RT, and 1.0 µg of total RNA. The reaction mixture was incubated for 1 h at 37 °C in a Tetrad thermocycler (MJ Research, Hercules, CA, USA). Before the reverse transcription reaction, total RNA was incubated at 65 °C for 5 min. qPCR reactions were performed using the 7300 Real Time PCR Systems apparatus (Applied Biosystems, Waltham, MA, USA). Cycling conditions were a 15 min initial denaturation or activation step, followed by 40 cycles (≈30 to 35 cycles for conventional RT-PCR) at 94 °C for 20 s, 59 °C for 20 s, and 72 °C for 20 s, with a final extension step of 5 min at 72 °C using the iCycler iQ Real-time Detection System (Bio-Rad Laboratories, Hercules, CA, USA). The 16S gene of *X. fastidiosa* was used as internal control. The threshold fluorescence was manually configured for all plates using SDS software (Applied Biosystems, Waltham, MA, USA). Analyses were performed from the Cycle threshold (Ct) using ∆∆Ct method.

### 4.5. Esterase and Lipase Activity Assays

The esterase activity of the secreted and total proteins (8.4 μg) was measured using the 4-methylumbelliferyl butyrate (4-MUB) substrate [94]. The blank for the secreted proteins was the concentrated PW culture medium and for the total cellular proteins was the BugBuster^®^ reagent used for sample preparation. Measurement of the fluorescent product obtained was performed after 30 min of reaction. Tributyrin (C4) (Sigma-Aldrich, USA) was used as substrate to evaluate the lipase activity of secreted proteins in a plate assay. The input of 1.2 mg of the concentrated samples referring to *X. fastidiosa* strains Fb7 and 9a5c with 6 and 10 days of growth were added in an orifice made in the medium (2% agarose, 1% tributyrin, 100 mM Tris-HCl pH 8.0 and 25 mM CaCl2). Concentrated PW culture medium was used as a negative control. The plate was incubated (28 °C for 5 days) to verify the formation of a halo of hydrolysis.

### 4.6. In Vivo Assay

Seeds of *Nicotiana tabacum* cultivar SR1 were propagated on Bioplant^®^ substrate (Bioplant Agrícola, Brazil) and kept in the greenhouse (average temperature of 23 °C and 65% humidity). At 55 days of growth, plants were inoculated with 20 µL of bacterial suspension of *X. fastidiosa* strains Fb7 or 9a5c (OD_600_ 0.05) at the base of the petiole of the third leaf (from bottom to top) of each plant which was previously perforated with a sterile needle to allow the absorption of the cultures by the xylem vessels. As negative control, 3 plants were inoculated with PW medium. Plants were monitored and symptoms evaluated after 30 days of inoculation.

## Figures and Tables

**Table 1:** Top 20 cellular extract proteins with higher abundance in *X. fastidiosa* subsp. *pauca* strain Fb7 over strain 9a5c of the same species.

Gene ID ^a^	Gene Category	Protein Description	Log _2_ FC (Fb7/9a5c)	*p*-Value	Exclusive Peptides	% Coverage
XF0872	IV.A.2	Outer membrane protein W	3.11	3.87 × 10^−6^	7	59
XF1742	VIII.A	Gluconolactonase	2.52	1.54 × 10^−6^	4	6
XF2677	I.A.2	L-ascorbate oxidase (Aoo)	2.36	1.40 × 10^−5^	8	27
XF2392	IV.A.2	Autolytic lysozyme	2.34	7.58 × 10^−7^	5	29
XF0156	III.C.3	Cysteine protease	1.94	3.59 × 10^−5^	11	42
XF1516	VII.F	Adhesin XadA1	1.85	2.53 × 10^−5^	7	10
XF0999	II.A.1	Argininosuccinate synthase	1.71	1.52 × 10^−6^	10	31
XF1123	IV.A.2	Outer membrane protein	1.59	1.68 × 10^−2^	3	44
XF1744	IX	Oxidoreductase	1.55	3.74 × 10^−6^	6	34
XF0619	VII.C	Periplasmic divalent cation tolerance	1.5	9.68 × 10^−3^	2	7
XF1282	III.C.3	Carboxypeptidase-related protein	1.45	3.02 × 10^−6^	3	8
XF0339	VIII.A	TonB-dependent receptor	1.44	9.91 × 10^−6^	9	9
XF1159	III.B.2	50S ribosomal protein L16	1.41	1.17 × 10^−2^	3	12
XF1745	VIII.A	Alpha/beta-Hydrolases	1.4	1.74 × 10^−4^	8	14
XF1741	VII.C	Daunorubicin C-13 ketoreductase	1.37	1.33 × 10^−4^	3	9
XF1165	III.B.2	30S ribosomal protein S14	1.33	4.85 × 10^−3^	2	27
XF2551	VIII.A	Dipeptidyl aminopeptidase protein 6	1.28	6.41 × 10^−5^	18	35
XF1537	III.B.2	50S ribosomal protein L13	1.21	2.54 × 10^−3^	2	18
XF1058	I.B.8	Uridylate kinase	1.12	1.80 × 10^−5^	2	12
XF0389	I.D	Two-component system, regulatory protein	1.11	5.88 × 10^−5^	2	37

^a^ Gene IDs and categories according to the *Xylella fastidiosa* 9a5c database http://aeg.lbi.ic.unicamp.br/xf, last accessed on July 2020.

**Table 2:** Top 20 cellular extract proteins with higher abundance in *X. fastidiosa* subsp. *pauca* strain 9a5c over strain Fb7 of the same species.

Gene ID	Gene Category ^a^	Protein Description	Log _2_ FC (Fb7/9a5c)	*p*-Value	Exclusive Peptides	% Coverage
XF2407	VII.C	Hemolysin-like RTX protein	−2.89	4.87 × 10^−6^	4	4
XF2200	VIII.B	Uncharacterized protein	−2.42	6.62 × 10^−4^	4	19
XF2341	III.C.2	Heat shock protein GrpE	−2.33	3.86 × 10^−4^	2	35
XF2041	VIII.B	Uncharacterized protein	−2.20	1.08 × 10^−6^	4	32
XF2542	IV.D	Fimbrial protein	−1.80	4.47 × 10^−4^	2	9
XF0550	VIII.A	Porin BtuB	−1.69	4.82 × 10^−4^	15	34
XF1026	III.C.3	Serine Protease PspB	−1.66	2.55 × 10^−4^	5	8
XF1981	VII.F	Adhesin XadA3	−1.54	5.43 × 10^−4^	14	24
XF2283	VIII.B	Metallo-β-lactamase family protein	−1.50	2.84 × 10^−4^	9	55
XF2307	VIII.B	Uncharacterized protein	−1.42	2.99 × 10^−5^	2	8
XF0082	IV.A.1	Chaperone protein precursor	−1.42	1.64 × 10^−3^	10	49
XF1297	II.C	Gluconolactonase precursor	−1.36	1.04 × 10^−5^	3	7
XF1843	I.D	Nitrogen regulatory protein P-II	−1.20	2.96 × 10^−4^	2	29
XF0942	I.C.7	Malate:quinone oxidoreductase	−1.19	1.40 × 10^−4^	6	30
XF0372	IV.D	Fimbrial assembly protein	−1.18	2.30 × 10^−3^	2	42
XF0611	IV.C	dTDP-glucose 4-6-dehydratase	−1.17	1.50 × 10^−5^	11	37
XF0078	IV.D	Fimbrial adhesin precursor	−1.10	1.29 × 10^−3^	6	3
XF0657	I.B.9	Alkaline phosphatase	−1.07	1.77 × 10^−2^	2	8
XF0816	III.C.3	Zn-dependent peptidase	−1.02	7.42 × 10^−4^	27	60
XF2093	VII.C	Precursor of drug resistance protein	−1.01	6.94 × 10^−3^	9	38

^a^ Gene IDs and categories according to the *Xylella fastidiosa* 9a5c database http://aeg.lbi.ic.unicamp.br/xf, last accessed on July 2020.

**Table 3:** Secreted proteins with differential abundance between *X. fastidiosa* strains Fb7 and 9a5c.

Gene ID ^a^	Gene Category	Protein Description	Log _2_ FC (Fb7/9a5c)	*p*-Value	Exclusive Peptides	% Coverage
**Proteins with Higher Abundance in Strain Fb7**						
XF1434	VIII.B	Cysteine/serine peptidase	1.88	2.73 × 10^−15^	2	14
XF0357	VIII.B	Lipase/Esterase LesA	1.70	2.50 × 10^−7^	5	24
XF1516	VII.F	Adhesin XadA1	1.66	1.20 × 10^−5^	8	14
XF0358	VIII.B	Lipase/Esterase LesB	0.46	3.49 × 10^−2^	2	18
**Proteins with Higher Abundance in Strain 9a5c**						
XF1026	III.C.3	Serine Protease PspB	−3.65	9.87 × 10^−22^	5	12
XF1577	VIII.B	Uncharacterized protein	−2.69	1.00 × 10^−3^	2	18
XF0668	VII.C	Hemolysin-like RTX protein	−2.33	6.65 × 10^−5^	3	8
XF2237	VIII.A	Outer membrane receptor	−2.16	1.00 × 10^−5^	5	16
XF2407	VII.C	Hemolysin-like RTX protein	−1.53	3.14 × 10^−1^	5	5
XF1287	VIII.B	Uncharacterized protein	−1.44	1.00 × 10^−5^	8	26
XF1547	IV.A.2	Membrane lipoprotein Lpp	−1.28	2.47 × 10^−6^	4	25
XF0267	III.C.3	Serine protease	−1.18	1.00 × 10^−3^	2	10
XF1011	VII.C	Hemolysin-like RTX protein	−1.13	4.84 × 10^−1^	2	5
XF0531	VIII.B	Cysteine/serine peptidase PrtA	−1.07	3.18 × 10^−1^	5	17
XF0343	IV.A.2	Outer membrane protein A	−0.58	1.08 × 10^−2^	5	12
XF1981	VII.F	Trimeric adhesin XadA3	−0.57	1.29 × 10^−2^	5	5

^a^ Gene IDs and categories according to the *Xylella fastidiosa* 9a5c database http://aeg.lbi.ic.unicamp.br/xf, last accessed on July 2020.

## Supplementary Materials

Supplementary materials can be found at https://www.mdpi.com/1422-0067/21/18/6769/s1.
