# Supplementary material for: Xylella fastidiosa subsp. pauca Strains Fb7 and 9a5c from Citrus Display Differential Behavior, Secretome, and Plant Virulence
Source: Int J Mol Sci. 2020 Sep 15;21(18):6769. doi: 10.3390/ijms21186769 (PMC7555403; doi:10.3390/ijms21186769)
Supplement: Supplementary file 1 [file ijms-21-06769-s001.zip › Supplementary Figures -- Souza et al.docx]

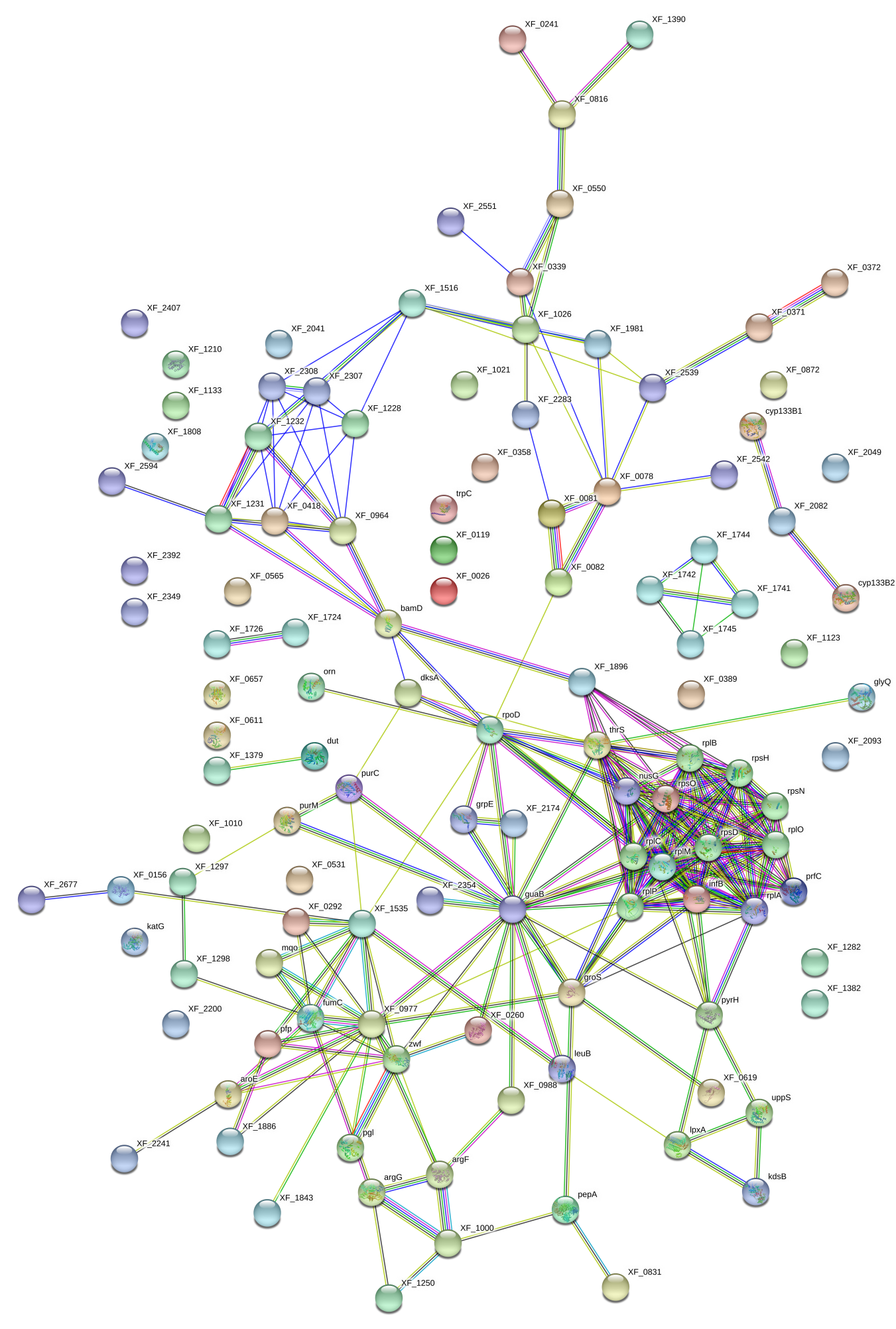


**Figure S1.** Network visualization of proteins with differential abundance between X. fastidiosa strains Fb7 and 9a5c, using STRING version 11.0, with 119 nodes and 254 edges. The average local clustering coefficient=0.483 and PPI enrichment p-value< 6.68e-07. The nodes represent proteins and the edges represent the predicted functional associations.


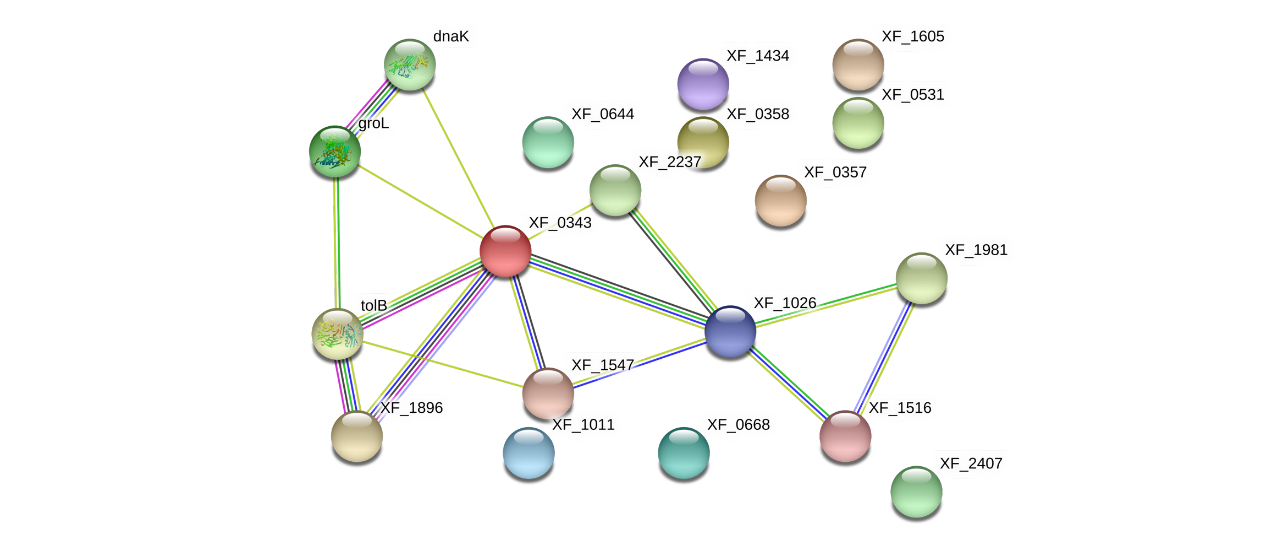


**Figure S2.** Network visualization of secreted proteins detected in X. fastidiosa culture supernatants using STRING version 11.0, with 19 nodes and 16 edges. The average local clustering coefficient=0.39 and PPI enrichment p-value< 0.00064. The nodes represent proteins and the edges represent the predicted functional associations.

| 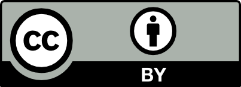 | © 2020 by the authors. Submitted for possible open access publication under the terms and conditions of the Creative Commons Attribution (CC BY) license (http://creativecommons.org/licenses/by/4.0/). |
| --- | --- |
